# Non-Host Defense Response in a Novel Arabidopsis-*Xanthomonas citri* subsp. *citri* Pathosystem

**DOI:** 10.1371/journal.pone.0031130

**Published:** 2012-01-27

**Authors:** Chuanfu An, Zhonglin Mou

**Affiliations:** Department of Microbiology and Cell Science, University of Florida, Gainesville, Florida, United States of America; University of Wisconsin-Milwaukee, United States of America

## Abstract

Citrus canker, caused by *Xanthomonas citri* subsp. *citri* (*Xcc*), is one of the most destructive diseases of citrus. Progress of breeding citrus canker-resistant varieties is modest due to limited resistant germplasm resources and lack of candidate genes for genetic manipulation. The objective of this study is to establish a novel heterologous pathosystem between *Xcc* and the well-established model plant *Arabidopsis thaliana* for defense mechanism dissection and resistance gene identification. Our results indicate that *Xcc* bacteria neither grow nor decline in Arabidopsis, but induce multiple defense responses including callose deposition, reactive oxygen species and salicylic aicd (SA) production, and defense gene expression, indicating that *Xcc* activates non-host resistance in Arabidopsis. Moreover, *Xcc*-induced defense gene expression is suppressed or attenuated in several well-characterized SA signaling mutants including *eds1*, *pad4*, *eds5*, *sid2*, and *npr1*. Interestingly, resistance to *Xcc* is compromised only in *eds1*, *pad4*, and *eds5*, but not in *sid2* and *npr1*. However, combining *sid2* and *npr1* in the *sid2npr1* double mutant compromises resistance to *Xcc*, suggesting genetic interactions likely exist between *SID2* and *NPR1* in the non-host resistance against *Xcc* in Arabidopsis. These results demonstrate that the SA signaling pathway plays a critical role in regulating non-host defense against *Xcc* in Arabidopsis and suggest that the SA signaling pathway genes may hold great potential for breeding citrus canker-resistant varieties through modern gene transfer technology.

## Introduction

Citrus canker is a devastating leaf, stem, and fruit spotting disease affecting many important citrus species such as grapefruit (*Citrus paradisis* Macf.), certain sweet oranges (*C. sinensis* (L.) Osbeck), Key lime (*C. aurantifolia* Swingle), and lemons (*C. limon* (L.) Burm. F.) [1]. It is caused by the bacterial pathogen *Xanthomonas citri* subsp. *citri* (*Xcc*) [2]. Although reduced quality and quantity of fresh and processed fruits have been causing great economic loss to the citrus industry, no efficient way has been found to control the disease. Currently, management of citrus canker largely relies on chemical control and agricultural practices [2]. Because of the economic and environmental concerns, developing resistant cultivars perhaps is the best long-term solution for the management [3]. However, limited resistant scion germplasm resources and their interfering with the expression of optimum traits related to fruit quality and production hamper developing canker-resistant citrus varieties through conventional breeding approach, not to mention its labor- and time-consuming characters [4]. In contrast, transgenic approach can quickly incorporate resistance into citrus without interfering with the expression of optimum varietal traits. Nevertheless, its accomplishment depends on the understanding of the molecular mechanisms of pathogenesis and the availability of target genes for manipulation [3]. As a highly heterozygous, polygenic species with limited genetic resources and a long juvenile period, functional analysis of citrus genes related to innate disease resistance is impaired, which consequently hinders the development of canker-resistant citrus cultivars using transgenic approach. The model plant *Arabidopsis thaliana* has been shown as a promising alternative for understanding plant defense mechanisms [5]–[7]. Transferring molecular technologies including genes involved in innate immunity from model plant to crops holds great potential for genetic improvement. In fact, several studies have already demonstrated its feasibility in the development of citrus disease resistant lines [3], [8].

In nature, plants are constantly challenged by a diverse range of microbes. However, for a certain plant species, only a few of these microbes are pathogenic. Resistance of an entire plant species against all strains of a pathogen that is able to infect other plant species is a phenomenon known as non-host resistance and dictates the most robust form of plant immunity [9]. Despite its great potential for providing crop plants with durable resistance, plant defense mechanisms underlying non-host resistance are not sufficiently understood [10]. Accumulating evidence has indicated that plant non-host resistance is composed of layers of defense responses [10]–[13]. To establish pathogenicity, pathogens need to enter plant tissue to obtain nutrients and counteract host defense. Phytopathogenic bacterium like *Pseudomonas syringae* enters the internal plant tissue through open stomata or wounds, whereas some fungal pathogens directly penetrate plant cell wall. Preformed physical and chemical barriers are thought to constitute the primary tranche of non-host defense mechanisms [9]. Several preformed (wax, cuticle layer, cell wall) and inducible barriers, such as papilla/callose [12], aliphatic isothiocyanates [14], indole glucosinolates [15], camalexin [16], and chloroplast-generated reactive oxygen species (ROS) [17], play important roles during non-host interactions. Two genes *AtGSNOR1* and *F3OGT*, related to *S*-nitrosothiol and anthocyanin biosynthesis, respectively, are thought important to non-host resistance [18], [19]. Studies on Arabidopsis against non-adapted phytopathogenic fungi barley powdery mildew (*Blumeria graminis* f.sp. *hordei*; *Bgh*) identified three genes involved in limiting *Bgh* penetration through two separate pathways. One involves an exocytosis pathway controlled by the PEN1 syntaxin and its working partners [20], [21] and the other requires the PEN2 myrosinase and the PEN3 ATP-binding cassette transporter [22], [23]. Inhibition of the actin skeletal function in combination with the *eds1* mutation severely compromises non-host resistance in Arabidopsis against wheat powdery mildew, which suggests that actin cytoskeleton is also involved in preinvasion non-host resistance [24]. Comparative gene expression profiling analyses revealed the similar defense responses between non-host resistance and gene-for-gene resistance in Arabidopsis [25], [26]. Moreover, among the non-host *Pseudomonas* bacteria-regulated genes, approximately 30% of them are also regulated by flg22, indicating a role of pathogen-associated molecular pattern (PAMP) signaling in non-host resistance [26]. Species- or family-level difference in PAMP recognition also suggests its association with non-host resistance [27]–[29]. Meanwhile, pathogen mutants lacking a functional PAMP were shown to gain at least partial virulence on non-host plants [30], [31]. These results indicate that PAMP recognition is another important non-host barrier. Furthermore, some genetic components involved in gene-for-gene host resistance were shown to function in post-invasive defense. Examples of *R* genes functioning in non-host resistance are few [32], [33]. However, several signaling components involved in gene-for-gene resistance have been identified from various pathosystems. Among them are the EDS1-PAD4-SAG101 complex [22], [23], the HSP90-SGT1-RAR1 complex [34]–[37], ADS1 [38], ARF1 [39], EDR1 [40], NDR1 [41], HSP70/HSP90 [42]–[44], and PAD3 [45]. In addition, a glycerol kinase-encoding gene *NHO1* is required for Arabidopsis resistance to heterologous bacterial pathogen *P. syringae* pv. *phaseolicola* and *P. syringae* pv. *tabaci* [46], [47].

Recent genetic and genomic studies also revealed the important role of salicylic acid (SA), jasmonic acid (JA), and ethylene (ET) for maintenance of non-host resistance in specific plant-microbe combinations [10]. Degradation of SA in Arabidopsis salicylate hydroxylase (*NahG*) transgenic plants confers susceptibility to the non-host bacterium *P. syringae* pv. *phaseolicola* NPS3121 [46]. Non-host resistance against the cowpea rust fungus *Uromyces vignae* requires accumulation of SA in Arabidopsis [48]. Non-host resistance of Arabidopsis to *Alternaria brassicicola* depends on JA, as *coi1* mutant is susceptible to fungal infection [49]. Moreover, tobacco plants impaired in ethylene perception are susceptible to a variety of soil-borne species in the genus of *Pythium* [50]. In another heterologous pathosystem between Arabidopsis and *Phakopsora pachyrhizi*, both SA and JA signaling pathway are involved [6]. The JA/ET pathway is also activated during non-host resistance to the hemibiotrophic potato pathogen, *Phytophthora infestans*, and the biotroph, *Blumeria graminis* f.sp. *hordei* in Arabidopsis [7]. A recent survey of a panel of Arabidopsis mutants, involved in gene-for-gene resistance, unveiled that both SA and JA/ET pathway contribute to post-invasive resistance against *Golovinomyces cichoracearum* UMSG1 [51].

In this study, we established a novel non-host pathosystem involving Arabidopsis and an economically important bacterial pathogen *Xcc*. By examination of a series of previously identified Arabidopsis mutants compromised in SA, JA, and ET defense signaling, several genetic components of the SA signaling pathway were found to play profound role in the *Xcc*-induced defense gene expression and the non-host resistance against *Xcc* in Arabidopsis. These results suggest that the Arabidopsis-*Xcc* pathosystem is highly valuable for identifying signaling components that positively regulate non-host resistance against *Xcc*. The SA signaling pathway genes pinpointed in this study could potentially be engineered into citrus to combat canker disease.

## Results

### *Xcc* is a non-host pathogen of Arabidopsis

**Figure 1:**
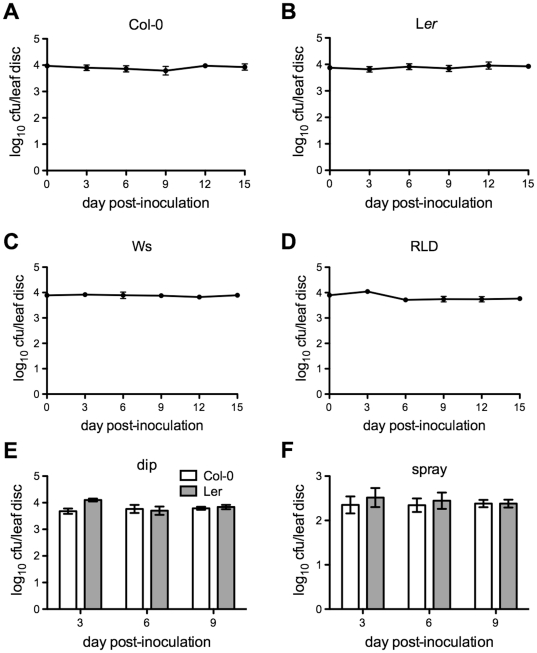
*Xcc* does not grow in Arabidopsis. (**A**) Growth of syringe-infiltrated *Xcc* in Col-0. (**B**) Growth of syringe-infiltrated *Xcc* in L*er*. (**C**) Growth of syringe-infiltrated *Xcc* in Ws. (**D**) Growth of syringe-infiltrated *Xcc* in RLD. (**E**) Growth of dip-inoculated *Xcc* in Col-0 and L*er*. (**F**) Growth of spray-inoculated *Xcc* in Col-0 and L*er*. Four-week-old plants were inoculated with *Xcc*. Bacterial suspensions with an OD_600_ of 0.002 and 0.02 were used for syringe infiltration and dip/spray inoculation, respectively. The *in planta* bacterial titers were determined on day 0, 3, 6, 9, 12, and 15 post-inoculation for syringe infiltration, and on day 3, 6, and 9 post-inoculation for dip and spray inoculation (cfu, colony-forming units). Data represent the mean of eight independent samples with standard deviation. The experiment was repeated twice with similar results.

To test whether the citrus canker bacterial pathogen *Xcc* could cause disease in Arabidopsis, we inoculated Arabidopsis plants with *Xcc* by syringe infiltration, dip, and spray inoculation methods. Leaf tissues were collected at different time points after inoculation to determine the *in planta* growth of *Xcc*. As shown in Figures 1A to 1D, syringe-infiltrated *Xcc* did not grow at all during the course of a relatively long-term (15 days) infection in four Arabidopsis ecotypes, Columbia (Col-0), Landsberg *erecta* (L*er*), Wassilewskija (Ws), and RLD. Similar result was observed from experiments using dip and spray inoculation methods. Bacterial number remained almost constant during 9 days post-inoculation (dpi) in both Col-0 and L*er* ecotypes (Figures 1E to 1F). We noticed that physical barriers blocked a large portion of bacteria coated on the surface of leaves by dip and spray inoculation methods. The concentration of the bacterial suspensions used for dip and spray inoculation was 100 fold higher than that used in syringe infiltration, but bacterial growth was at the same level (dip) as or less (spray) than that observed in the inoculation by syringe infiltration (Figures 1E to 1F). Interestingly, the numbers of *Xcc* bacteria did not decline during the course of infection, indicating that *Xcc* is able to survive for a long period of time in Arabidopsis. Thus, *Xcc* is a non-host pathogen of Arabidopsis. Furthermore, we did not observe any visible symptoms associated with the infection, suggesting that *Xcc* may induce type I non-host resistance in Arabidopsis [10].

### *Xcc* activates a multilayered defense response in Arabidopsis

**Figure 2:**
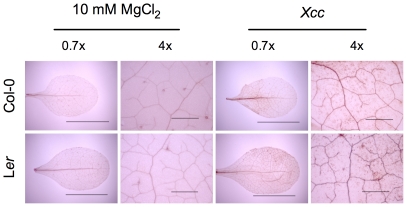
*Xcc* induces ROS accumulation in Arabidopsis. Four-week-old Arabidopsis plants were syringe-infiltrated with *Xcc* (OD_600_ = 0.02) or mock control (10 mM MgCl_2_). At 4 hpi, infiltrated leaves were excised and stained with DAB (3,3′-diaminobenzidine tetrahydrochloride). The presence of ROS (mainly hydrogen peroxide) caused polymerization of DAB, yielding a reddish-brown color. Tissue was examined under a Leica MEIJI scope. Representative images shown here came from 24 leaves from 12 independent plants. Bars represent 1 cm and 1 mm in images magnified 0.7 and 4 folds, respectively.

**Figure 3:**
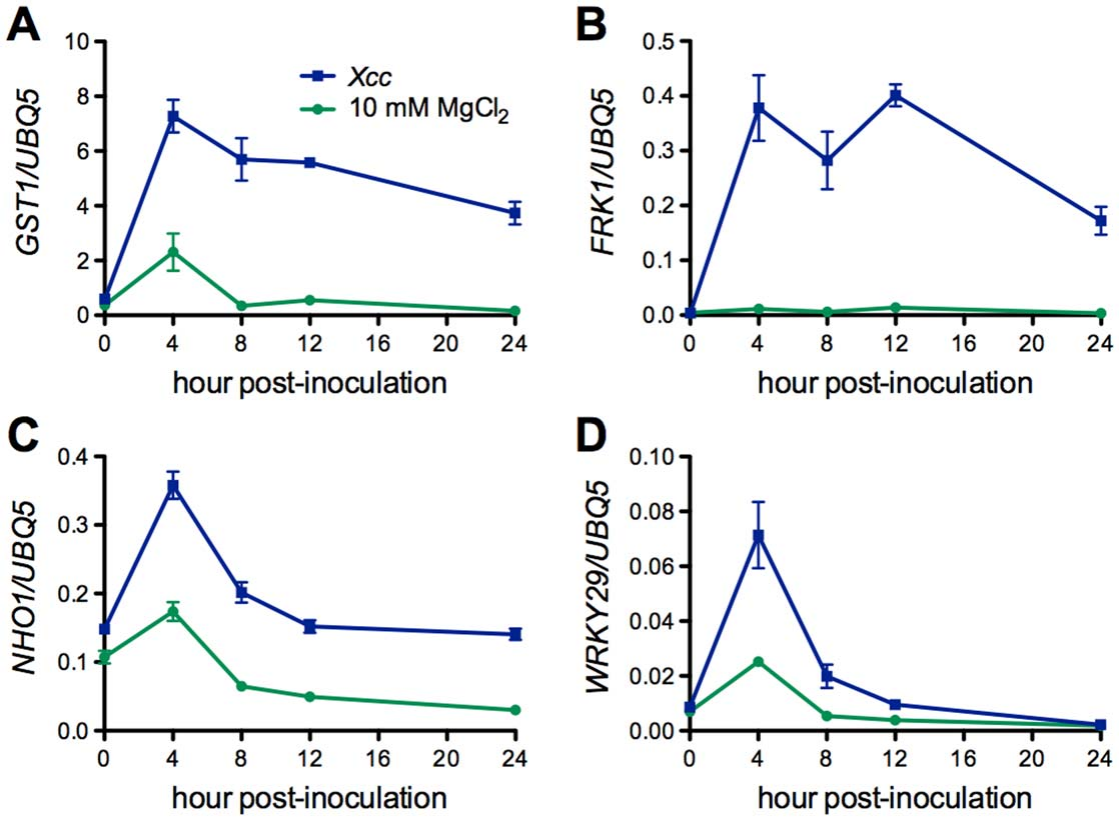
*Xcc* activates both ROS- and flg22-inducible early response genes in Arabidopsis. (**A**) Expression of *GST1*. (**B**) Expression of *FRK1*. (**C**) Expression of *NHO1*. (**D**) Expression of *WRKY29*. Four-week-old Col-0 plants were inoculated with *Xcc* (OD_600_ = 0.02) or mock-treated with 10 mM MgCl_2_. Leaf samples were collected at different time points (0, 4, 8, 12, and 24 hpi) for total RNA isolation and gene expression analysis using RT-qPCR. Expression levels were normalized against constitutively expressed *UBQ5*. *GST1* is a marker gene for the engagement of ROS-dependent defense. *FRK1*, *NHO1*, and *WRKY29* are flg22-inducible genes. Data represent the mean of three biological replicates with standard deviation. The experiment was repeated twice with similar results.

**Figure 4:**
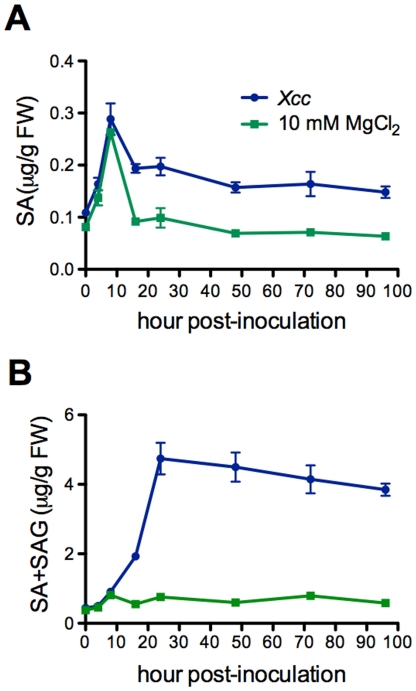
*Xcc* induces SA production in Arabidopsis. (**A**) Free SA levels. (**B**) Total SA (SA+SAG) levels. Leaves of wild-type Col-0 plants were inoculated with *Xcc* (OD_600_ = 0.02) or treated with 10 mM MgCl_2_. The inoculated leaves were collected at different time points (0, 4, 8, 16, 24, 48, 72, and 96 hpi) for SA measurement by HPLC. Data represent the mean of four independent samples with standard deviation. The experiment was repeated twice with similar results.

**Figure 5:**
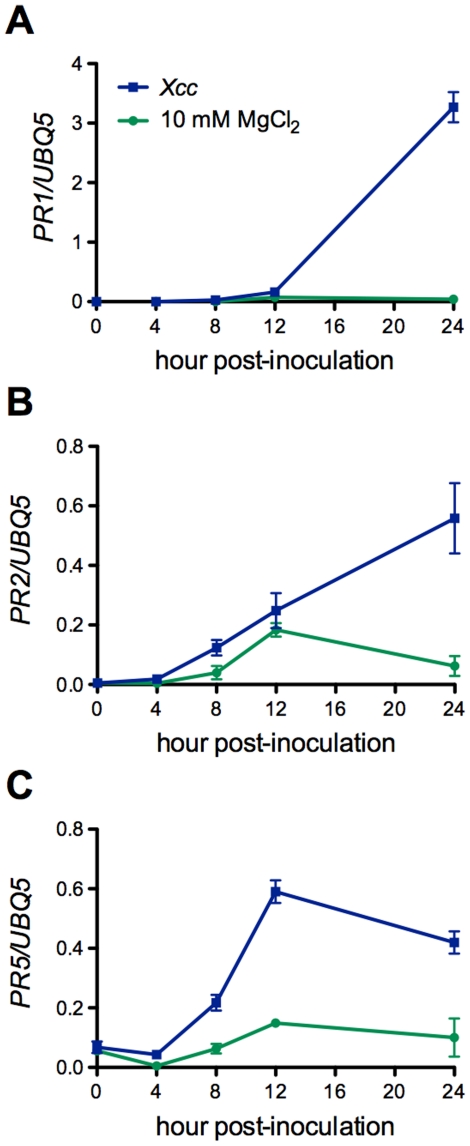
*Xcc* induces *PR* gene expression in Arabidopsis. (**A**) Expression of *PR1*. (**B**) Expression of *PR2*. (**C**) Expression of *PR5*. Four-week-old Col-0 leaves were inoculated with *Xcc* (OD_600_ = 0.02) or mock-treated with 10 mM MgCl_2_. Leaf samples were collected at different time points (0, 4, 8, 12, and 24 hpi) for total RNA isolation and gene expression analysis using RT-qPCR. Expression levels were normalized against constitutively expressed *UBQ5*. Data represent the mean of three biological replicates with standard deviation. The experiment was repeated twice with similar results.

The involvement of ROS in both host and non-host response has been extensively studied [52]–[55]. To assay the role of ROS in the Arabidopsis-*Xcc* interaction, we examined ROS accumulation by DAB (3,3′-diaminobenzidine tetrahydrochloride) staining and monitored the dynamic expression of *GST1*, a marker gene for the engagement of ROS-dependent defense [56], in *Xcc*-infected Arabidopsis leaves. In both Col-0 and L*er* ecotypes, ROS accumulation was detected at 4 hours post-inoculation (hpi) (Figure 2). Further, *GST1* expression peaked at 4 hpi, and then gradually decreased (Figure 3A). Together, these results indicate that ROS may be a non-host defense component in the Arabidopsis-*Xcc* interaction. Moreover, PAMP-induced early response genes appeared to participate in the non-host defense response in the Arabidopsis-*Xcc* pathosystem. Three flg22-inducible genes, *FRK1*, *NHO1*, and *WRKY29* [31], [57], were significantly activated by *Xcc* infection (Figures 3B to 3D). Expression of these genes reached the highest level at 4 hpi and decreased afterward, except that the expression levels of *FRK1* maintained high from 4 to 12 hpi (Figure 3B). To test whether SA is involved in the non-host interaction between Arabidopsis and *Xcc*, we measured SA levels in *Xcc*-infected leaf tissues. As shown in Figure 4A, there was a slight increase in free SA levels at 8 hpi, which may be caused by the infiltration, as free SA levels also increased slightly in the MgCl_2_-treated leaf tissues. However, total SA levels in the *Xcc*-infected leaf tissues increased significantly, reaching the highest at 24 hpi and staying at the plateau till the end of the experiment (96 hpi) (Figure 4B). We further tested if *Xcc* infection could trigger the expression of SA-dependent pathogenesis-related (*PR*) genes [58]. Compared with the mock treatment (10 mM MgCl_2_), *Xcc* inoculation induced the expression of *PR1*, *PR2*, and *PR5* in Col-0 plants (Figures 5A to 5C). In this specific interaction, *PR2* and *PR5* were induced earlier (4–8 hpi) than *PR1* (12 hpi), whereas *PR1* appeared to be induced to a higher level than *PR2* and *PR5*. These results indicate that the well-defined SA signaling pathway is activated during the Arabidopsis-*Xcc* interaction.

### Non-host resistance to *Xcc* is compromised in several SA signaling mutants

**Figure 6:**
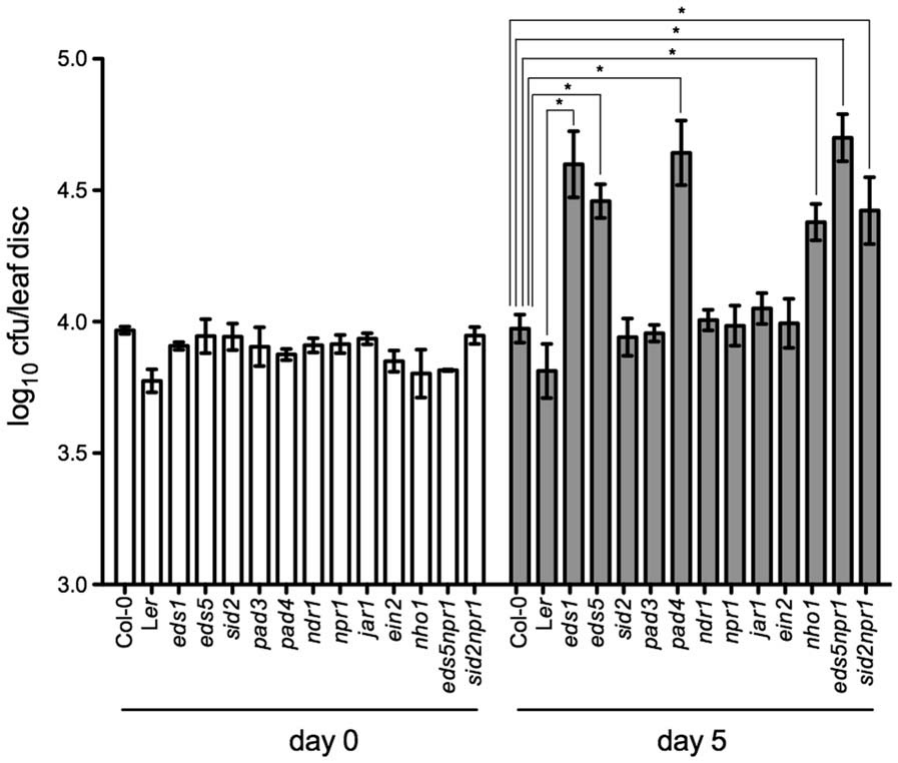
Growth of *Xcc* in several Arabidopsis SA, JA, and ET singling mutants. Leaves of four-week-old plants were inoculated with *Xcc* (OD_600_ = 0.002). The *in planta* bacterial titers were determined immediately (day 0) or on day 5 post-inoculation (cfu, colony-forming units). Data represent the mean of eight independent samples with standard deviation. *Xcc* grew significantly more in *eds5*, *pad4*, *nho1*, *eds5npr1*, and *sid2npr1* than in the wild-type Col-0 plants (*P<0.01, 0.001, 0.001, 0.001, and 0.05, respectively). Similarly, *Xcc* grew significantly more in *eds1* than in the wild-type L*er* plants (*P<0.01). The experiment was repeated three times with similar results.

To reveal whether the SA signaling pathway contributes to the non-host resistance against *Xcc* in Arabidopsis, we quantified the growth of *Xcc* in a series of single or double mutants related to SA signaling (*npr1*, *eds1*, *eds5*, *sid2*, *pad4*, *ndr1*, *eds5npr1*, and *sid2npr1*). A JA signaling mutant (*jar1*), an ET signaling mutant (*ein2*), a camalexin mutant (*pad3*), and the non-host defense mutant, *nho1*, which was identified in the Arabidopsis-*Pseudomonas syringae* pv. *phaseolicola* interaction [46], [47], were also included in the experiment. As shown in Figure 6, *Xcc* did not grow in *npr1*, *sid2*, *pad3*, *ndr1*, *ein2*, and *jar1*, but had a significant growth (∼5 fold) in *nho1*, *eds1*, *eds5*, and *pad4*. Interestingly, there was a significant growth of *Xcc* in the double mutant *sid2npr1*, though *Xcc* did not grow in either *npr1* or *sid2* single mutant. The growth of *Xcc* in *eds5npr1* was also higher than in the *eds5* single mutant. These results suggest that *NPR1* may genetically interact with *SID2* and *EDS5* in regulating non-host resistance against *Xcc* in Arabidopsis. More importantly, all mutants except *nho1* with enhanced susceptibility to *Xcc* are related to SA signaling, demonstrating that the SA signaling pathway plays an important role in the non-host resistance against *Xcc* in Arabidopsis.

### *Xcc*-induced callose deposition is not altered in the SA signaling mutants

**Figure 7:**
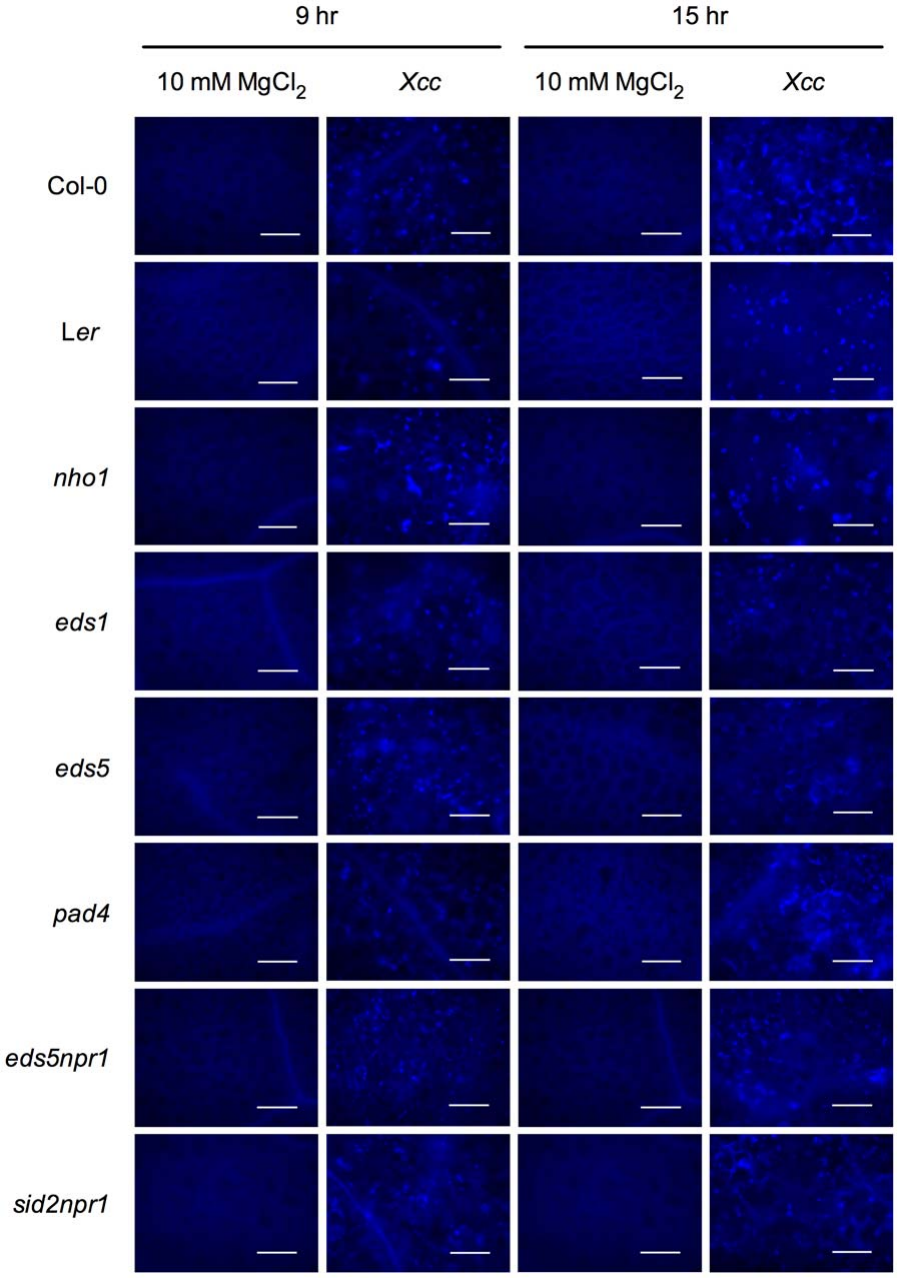
*Xcc*-induced callose deposition is not changed in the SA signaling mutants. Four-week-old Arabidopsis plants were inoculated with *Xcc* (OD_600_ = 0.2) or mock-treated with 10 mM MgCl_2_. At 9 and 15 hpi, inoculated leaves were excised and stained with aniline blue. Fluorescence was observed using an Olympus BH-2 epifluorescent microscope. No significant differences were detected among wild type (Col-0 and L*er*) and the mutant plants. Representative images shown here came from 24 leaves from 12 independent plants. Bars represent 100 µm.

To characterize if callose deposition contributes to the susceptibility of the SA signaling mutants to *Xcc*, callose staining was performed on *Xcc*-infected Arabidopsis leaves. After fixation, rehydration, and washing, translucent leaves were stained with aniline blue and examined by epifluorescent illumination. Consistent with the observation that mutations of *sid2*, encoding the SA biosynthesis gene *ICS1* [59], had no effect on callose production [60], [61], callose deposition was induced by *Xcc* infection in all the SA signaling mutants susceptible to *Xcc* (Figure 7), indicating that the SA signaling pathway is not required for *Xcc*-induced callose deposition. Moreover, no quantitative differences in callose deposition were detected among the mutants and the wild-type plants. Therefore, callose deposition did not contribute to the observed susceptibility of the SA signaling mutants to *Xcc*.

### *Xcc*-induced expression of early defense-response genes is decreased in the SA signaling mutants

**Figure 8:**
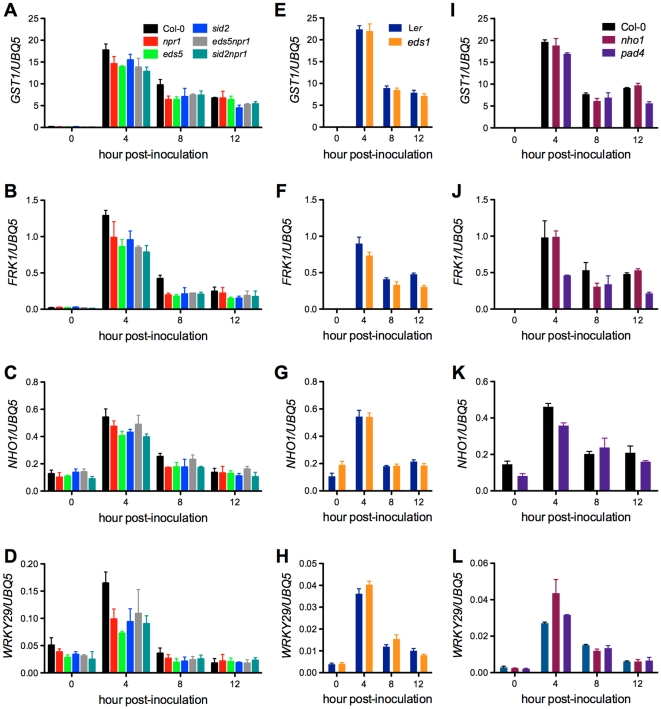
Expression of early response genes in the *Xcc* susceptible mutants. (**A** to **D**) Expression of *GST1*, *FRK1*, *NHO1*, and *WRKY29* in *npr1*, *eds5*, *sid2*, *eds5npr1*, and *sid2npr1*. (**E** to **H**) Expression of *GST1*, *FRK1*, *NHO1*, and *WRKY29* in *eds1*. (**I** to **L**) Expression of *GST1*, *FRK1*, *NHO1*, and *WRKY29* in *nho1* and *pad4*. Four-week-old plants were inoculated with *Xcc* (OD_600_ = 0.02). Leaf tissues were collected at different time points (0, 4, 8, and 12 hpi) for total RNA isolation and gene expression analysis using RT-qPCR. Expression levels were normalized against constitutively expressed *UBQ5*. Data represent the mean of three biological replicates with standard deviation. Mutant *eds1* is in L*er* genetic background, whereas others (*nho1*, *eds5*, *pad4*, *sid2*, *npr1*, *eds5npr1*, and *sid2npr1*) are in Col-0 genetic background. The experiment was repeated twice with similar results.

PAMP detection is an important component of non-host resistance in plants and serves as an early warning system for the presence of potential pathogens [31], [62]. Similarly, oxidative burst is another quick defense response after both host and non-host pathogen recognition [17]. To determine whether PAMP- or ROS-dependent early responses contribute to the observed susceptibility of the SA signaling mutants to *Xcc*, we compared the expression levels of *GST1*, *FRK1*, *NHO1*, and *WRKY29* after *Xcc* infection [31], [56], [57]. Expression levels of the four genes were generally lower in the SA signaling mutants than in the wild-type plants (Figure 8). The non-host defense mutation *nho1* only slightly lowered the expression of *GST1*, *NHO1*, and *WRKY29* at 8 hpi (Figures 8I, 8J and 8L). Although *npr1* and *sid2* did not allow *Xcc* growth, expression of the four genes was similarly inhibited in *npr1* and *sid2* as in other susceptible SA signaling mutants.

### *Xcc*-induced *PR* gene expression is suppressed in the SA signaling mutants

**Figure 9:**
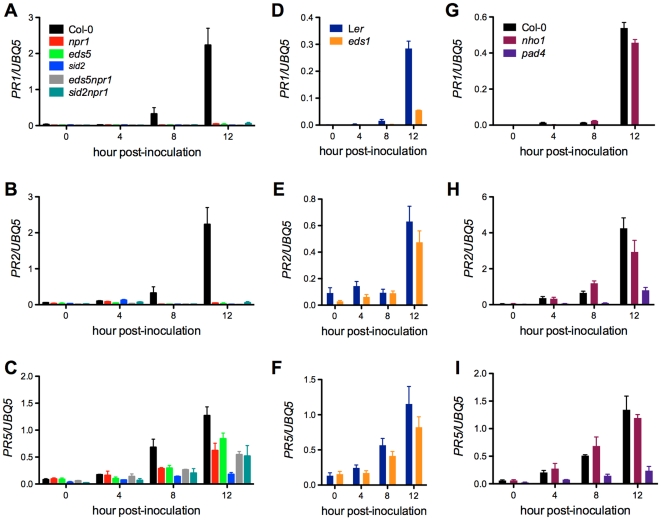
Expression of *PR* genes in the *Xcc* susceptible mutants. (**A** to **C**) Expression of *PR1*, *PR2*, and *PR5* in *npr1*, *eds5*, *sid2*, *eds5npr1*, and *sid2npr1*. (**D** to **F**) Expression of *PR1*, *PR2*, and *PR5* in *eds1*. (**G** to **I**) Expression of *PR1*, *PR2*, and *PR5* in *nho1* and *pad4*. The experiment was performed as in Figure 7. Expression was normalized against constitutively expressed *UBQ5*. Data represent the mean of three biological replicates with standard deviation. Mutant *eds1* is in L*er* genetic background, whereas others (*nho1*, *eds5*, *pad4*, *sid2*, *npr1*, *eds5npr1*, and *sid2npr1*) are in Col-0 genetic background. *Xcc*-induced expression of *PR1*, *PR2*, and *PR5* was dramatically inhibited in all the tested mutants except *nho1*. The experiment was repeated twice with similar results.

In Arabidopsis, *PR* gene expression is not only tightly correlated with resistance to host pathogens [58], but also related to non-host resistance [5], [46]. The finding that *Xcc* induces SA-dependent defense response prompted us to test how *Xcc*-induced *PR* gene expression is regulated in the SA signaling mutants susceptible to *Xcc*. Gene expression analysis revealed that *Xcc*-induced *PR* gene expression was significantly suppressed in the three susceptible single mutants (*eds1*, *eds5*, and *pad4*) and the two susceptible double mutants (*eds5npr1* and *sid2npr1*) (Figure 9). Although *Xcc*-induced *PR* gene expression was impaired in *npr1* and *sid2* (Figures 9A to 9C), both *npr1* and *sid2* were not more susceptible to *Xcc* than wild type. *Xcc*-induced *PR* gene expression in *nho1* was also decreased, but to a much lesser extent compared with that in the SA signaling mutants (Figures 9G to 9I).

## Discussion

Resistance of an entire plant species to all isolates of a microbial species is referred to as non-host resistance [9]. It is thought to comprise a variety of distinct mechanisms involved in layers of diverse processes [11]. More complicatedly, non-host resistance varies among different pathosystems [63]. In the present study, we elected to employ *Arabidopsis thaliana* as a model for understanding non-host resistance to the economically important bacterial pathogen *Xcc*, which causes canker disease to several citrus species. On the challenging road to disease, presence of preformed barriers is the first line of plant defense, which include cell wall, antimicrobial enzymes, and secondary metabolites [9], [64], [65]. When a non-host pathogen manages to overcome constitutive defensive layers, it becomes subject to the recognition at the plasma membrane of the plant cells. Elicitors released by host or non-host pathogens can activate PAMP recognition with the involvement of leucine-rich repeat (LRR)-receptor kinases and a MAP kinase cascade, which eventually leads to basal resistance [57], [66]. Inducible defense responses in non-host plants also include synthesis and accumulation of ROS, papillary callose, and phytoalexins, with or without formation of the hypersensitive response (HR) [67]. The last option of the obstacles to the non-host pathogen is the resistance mediated by independently and simultaneously activating pairs of pathogen *avr* and plant *R* gene i.e. gene-for-gene resistance [13]. Therefore, similar defense mechanisms exist between host and non-host interactions [25], [26]. Here, we showed that *Xcc* could also activate multilayered defense responses in Arabidopsis, which include ROS induction (Figures 2 and 3A), callose deposition (Figure 7), and PAMP- and SA-induced defense gene expression (Figures 3 and 5). The involvement of ROS in non-host response has been found in various plant-pathogen systems [52]–[55]. However, ROS production does not always lead to HR. Several lines of evidence from various plant species suggest that the sources of ROS are different during non-host response and during the HR, but these sources may interact with each other [68]–[70]. Interaction between Arabidopsis and *Xcc* induces ROS production (Figure 2), but not visible HR. Similar response was found in the interaction between Arabidopsis and the soybean pathogen *Pseudomonas syringae* pv. *glycinea* or the bean pathogen *P. syringae* pv. *phaseolicola* [5]. Nevertheless, several species of pathogens from genus *Xanthomonas* cause type II (with HR) non-host resistance in different hetergologous interactions [10]. The deposition of a linear ß-1,3-glucan polymer, callose, in response to pathogen attacking/wounding stresses is a basic defense mechanism that enables the plant to arrest pathogen proliferation by reinforcing the cell wall [71]–[74]. During the non-host interaction between Arabidopsis and *Xcc*, we found that callose is strongly induced (Figure 7). However, as a general response to bio/abiotic stresses, callose itself is just one component of multilayered non-host defense mechanism and needs coordination with others [12], [75]. No difference in callsoe deposition was observed between *Xcc* susceptible SA signaling mutants and wild-type plants. It is thus unclear whether callose deposition contributes to the non-host resistance to *Xcc* in Arabidopsis. Previous studies have established that PMAP-triggered defense response plays an important role in non-host resistance [31], [67]. Three flg22-inducible genes, *FRK1*, *NHO1*, and *WRKY29*, were a group of early response genes induced by *Xcc* (Figures 3B to 3D), suggesting an important role of flagellin-induced innate immunity in this pathosystem. Another component of the multilayered defense barriers of the non-host Arabidopsis plant to *Xcc* is the SA-mediated defense response with the activation of *PR* genes (Figures 4 and 5). Infection of Arabidopsis plants with *P. syringae* pv. *phaseolicola* NPS3121 induces SA accumulation and non-host resistance [76]. In contrast, removal of SA by a *NahG* transgene confers susceptibility to the same non-host pathogen [46]. In addition, induction of *PR* genes upon infection of different non-host pathogens has been found in multiple plant species [5], [12], [41], [46], [77]. Therefore, it seems clear that SA signaling is involved in non-host resistance. However, there is an observation suggesting that catechol (degradation product of SA), instead of SA, is responsible for the loss of non-host resistance in Arabidopsis *NahG* plants due to the resistant phenotype of several mutants with defects in SA signaling [76]. This divergence may be explained by variations of pathogen growth experiments and different criteria for non-host resistance/susceptibility among laboratories.

To determine if the known SA-dependent and/or JA/ET-dependent signaling pathway are involved in non-host resistance of Arabidopsis against *Xcc*, a group of Arabidopsis mutants that are impaired in SA or JA/ET signaling were employed for bacterial growth examination. In addition to EDS1 and PAD4, two components of the EDS1-PAD4-SAG101 signaling complex, which have been revealed to function in SA-mediated non-host resistance in multiple pathosystems [22], [23], non-host resistance against *Xcc* was abolished in the absence of a functional *EDS5* in Arabidopsis (Figure 6). Unlike previous reports, we did not observe *Xcc* growth in mutant *jar1* [48], *ein2* [78], *npr1* [48], *pad3* [45], *ndr1* [41], and *sid2* [48], [51], which provided another line of evidence that the mechanisms of non-host resistance vary among pathosystems [25]. Interestingly, mutation of *npr1* or *sid2* alone does not confer susceptibility to *Xcc*; however, combining both mutations together compromises non-host resistance to *Xcc* in the double mutant *sid2npr1* (Figure 6). This result reveled an undefined interaction between *NPR1* and *SID2* in the non-host resistance against *Xcc*. Similarly, *NPR1* may also interact with *EDS5* in the Arabidopsis-*Xcc* pathosystem, since the double mutant *eds5npr1* is more susceptible to *Xcc* than *eds5* (Figure 6). Thus, *NPR1*, a master regulator of multiple immune responses, may also play an important role in non-host resistance *via* either direct [48] or indirect ways (as shown here).

In the *Xcc* susceptible SA signaling mutants, induction of both early response genes (ROS- and flg22-inducible) and *PR* genes is inhibited in response to *Xcc* infection (Figures 8 and 9). However, decreased expression of these defense readouts in certain mutants such as *npr1* and *sid2* does not constitute susceptibility to *Xcc* (Figure 6). Furthermore, no difference in the expression of the defense genes was found between *sid2* or *npr1* and the susceptible double mutant *sid2npr1* (Figures 8 and 9). Similarly, although *eds5npr1* is more susceptible to *Xcc* than *eds5* and *npr1*, induction of the defense genes is comparable in all three mutants (Figures 8 and 9). Clearly, non-host resistance against *Xcc* is determined by the interaction of multiple defense mechanisms. Although some mutations could suppress certain *Xcc*-induced defense readouts, whether they could promote *Xcc* growth depends on their position in the complex defense network [5]. We identified *EDS1*, *EDS5*, *PAD4*, and *NHO1* as crucial components in the interaction of Arabidopsis and *Xcc*. Mutations in any of these genes lead to *Xcc* growth in Arabidopsis. In contrast, other genes, like *NPR1* and *SID2*, may genetically interact with each other in non-host resistance against *Xcc*.

In this study, we characterized a novel non-host pathosystem involving Arabidopsis and the citrus canker-causing bacterial pathogen *Xcc*. Using genetic and molecular analysis, we obtained an overview of the multilayered defense responses associated with the non-host resistance against *Xcc* in Arabidopsis. The pathosystem described here not only offered an excellent tool for improving our understanding of non-host defense response but also shed light on developing disease-resistant citrus varieties by transferring defense knowledge from model plants. The feasibility of this strategy has been proved by a recent study showing that overexpression of the Arabidopsis *NPR1* gene in citrus increases resistance to citrus canker [3]. Using non-host resistance for crop improvement has attracted much attention because this form of immunity is durable and can provide protection against all isolates of a pathogen species [63]. An excellent example is that a non-host wheat stripe rust resistance gene *Yr9* from rye played a very important role in controlling wheat stripe rust worldwide for a long time [79], [80]. Genes revealed to play important role in the non-host interaction between Arabidopsis and *Xcc* hold great potential for breeding canker-resistant citrus varieties through modern gene transfer technology.

## Materials and Methods

### Plant materials, growth, and pathogen infection

The wild-type plants used were *Arabidopsis thaliana* (L.) Heynh. Columbia (Col-0), Landsberg *erecta* (L*er*), Wassilewskija (Ws), and RLD ecotypes, and the mutant alleles used were *npr1-1* [81], *eds1-2* [82], *eds5-1* [83], *sid2-1* [84], *pad3-1* [85], *pad4-1* [86], *ndr1-1* [87], *ein2-2* [88], and *jar1-1* [89]. Two double mutants, *eds5npr1* and *sid2npr1*, were generated by crossing *npr1-1* with *eds5-1* and *sid2-1*, respectively. The *nho1* mutant seeds were obtained from the Arabidopsis Biological Resource Center (ABRC) (SALK_067205) [46], [47], and homozygous T-DNA insertion plants were identified by PCR. All mutants are in Col-0 genetic background except *eds1-2*, which is in L*er* background. Plants were grown in Metromix MVP soil (Bellevue, WA) under a 16 hr light/8 hr dark photoperiod at ∼22°C. Four-week-old plants were inoculated with *Xcc* strain 306 by syringe infiltration [90], dip or spray inoculation [91]. For dip and spray inoculation, plants were kept at high humidity by a plastic dome for two days. After inoculation, eight leaves were collected from different plants at each time point for each genotype to determine *in planta* growth of *Xcc*.

### Bacterial culture

The citrus canker causative bacterium *Xcc* strain 306 was obtained from Dr. James Graham (Citrus Research and Education Center, University of Florida) [92]. The bacteria were streaked from a glycerol stock onto Nutrient Broth (NB)-agar plate containing 20 µg/ml rifampin. After cultured at 30°C for two days, a single colony was picked up and cultured overnight in 3 mL liquid NB/rifampin at 30°C with a rotational speed of 220 rpm. For syringe infiltration inoculation, the 3 mL overnight culture was directly used. For dip and spray inoculation, the 3 mL overnight was added to 500 mL liquid medium and further cultured overnight. Bacterial cells were spun down and the pellet was resuspended in 10 mM MgCl_2_ to desired OD_600_ values for different experiments: 0.002 for syringe infiltration inoculation, 0.02 for gene expression, SA quantification, and ROS staining, and 0.2 for dip and spray inoculation and callose staining.

### ROS and callose staining

Four-week-old plants were syringe-infiltrated with a suspension of *Xcc* bacteria or mock control (10 mM MgCl_2_). Twenty-four leaves from 12 plants were used for both staining purposes. DAB (3,3′-diaminobenzidine tetrahydrochloride) staining for ROS (mainly hydrogen peroxide) was reported elsewhere [93]. Leaf samples were excised at 4 hpi for DAB staining and destained leaf samples were examined for reddish-brown coloration under a Leica MEIJI scope (Wetzlar, Germany). For callose staining, leaf samples were collected at 9 and 15 hpi, fixed in 3∶1 ethanol-to-glacial acetic acid under brief vacuum and then on a shaker with several changes of fixative until leaves appeared slightly translucent. Then the leaf samples were rehydrated sequentially in 70% and 50% ethanol solution each for over two hours. After washing twice with water, the leaf samples were left in water overnight on a shaker. The leaf samples were then incubated in 150 mM K_2_HPO_4_ (pH 9.5) solution containing 0.01% aniline blue for over four hours [94]. The leaf samples were mounted on slides with 50% glycerol and detected with an Olympus BH-2 epifluorescent microscope (Shinjuku, Tokyo, Japan) under UV illumination with broadband DAP filter set (excitation filter 390 nm, dichroic mirror 420 nm, emission filter 460 nm).

### RNA extraction and real-time quantitative PCR analysis

We used syringe infiltration as bacterial inoculation method for gene expression analyses. RNA extraction followed the protocol described previously [95]. Briefly, 100 mg leaf tissues infected with *Xcc* were ground to fine powders in liquid nitrogen with a Spex SamplePrep 2000 Geno/Grinder (OPS Diagnostics, Lebanon, NJ) and extracted with 80°C pre-warmed water-saturated phenol and RAPD buffer (100 mM LiCl, 100 mM Tris pH 8.0, 100 mM EDTA, and 1% SDS). The aqueous phase was extracted with chloroform, and the resulting aqueous phase was precipitated with ethanol at −80°C for one hour. RNA was pelleted by centrifugation, washed once with 80% ethanol, dried on ice, and suspended in 40 µl DEPC-treated water. RNA quality was checked with formaldehyde-agarose gel electrophoresis, and RNA concentration was measured with a NanoDrop 2000 spectrometer (Thermo Scientific, Wilmington, DE). For reverse transcription, total RNA was treated with DNase I (Ambion, Austin, TX) at 37°C for 30 minutes. After inactivation of the DNase, 2 µg RNA was reverse transcribed by M-MLV Reverse Transcriptase first-strand synthesis system (Promga, Madison, WI). The resulting cDNA products were diluted 20 folds with water, and 2.5 µl of the diluted cDNA products were used for quantitative real-time PCR analysis in an Mx3005P qPCR system (Agilent Technologies, Santa Clara, CA). All qPCR reactions were performed in duplicate using the SYBR Green protocol (Applied Biosystems, Foster City, CA) with a 12.5 µl reaction volume and a 0.4 µM primer concentration. The amplification condition was 95°C for 10 min followed by 40 cycles of 94°C for 30 sec, 55°C for 1 min, and 72°C for 1 min. PCR specificity was checked by dissociation analysis after the run was completed. Relative mRNA abundance to the reference gene *UBQ5* was calculated according to the delta Ct method. Primers for amplification of *UBQ5*, *PR1*, *PR2*, and *PR5* were reported elsewhere [96]. Primer sequences of the other genes are listed below: *GST1* (*qGST1F*: 5′-GTTCCAGCCTTTGAAGATGG-3′; *qGST1R*: 5′-TCCTTGCCAGTTGAGAGAAG-3′), *FRK1* (*qFRK1F1*: 5′-TGAGTCAGGTCGTTATGGAG-3′; *qFRK1R1*: 5′-ATTCACTACCTTGCTCGAGG-3′), *NHO1* (*qNHO1F*: 5′-CCACAGCTAACAACCTTCTG-3′; *qNHO1R*: 5′-AGAGAATCTGTTGTCGGACG-3′), and *WRKY29* (*qWRKY29F*: 5′-AGAGAATCTGTTGTCGGACG-3′; *qWRKY29R*: 5′-ACACCCTTTTGAGCTACTGC-3′).

### Salicylic acid quantification

Leaf tissues syringe-infiltrated with *Xcc* or mock control (10 mM MgCl_2_) were collected at the indicated time points. Measurement of both free and total SA was performed by HPLC method as reported [97].

### Statistical analysis

Data analysis tool *t*-TEST in Excel of Microsoft Office 2007 for Macintosh was used for all statistical analyses.

## References

[1] Gottwald TR, Graham JH, Civerolo EL, Barrett HC, Hearn CJ (1993). Differential host range reaction of citrus and citrus relatives to citrus canker and citrus bacterial spot determined by leaf mesophyll susceptibility.. Plant Dis.

[2] Graham JH, Leite RP (2004). Lack of control of citrus canker by induced systemic resistance compounds.. Plant Dis.

[3] Zhang X, Francis MI, Dawson WO, Graham JH, Orbović V (2010). Over-expression of the Arabidopsis *NPR1* gene in citrus increases resistance to citrus canker.. Eur J Plant Pathol.

[4] Viloria Z, Drouilard DL, Grahm JH, Grosser JW (2004). Screening triploid hybrids of ‘Lakeland’ Limequat for resistance to citrus canker.. Plant Dis.

[5] Mishina TE, Zeier J (2007). Bacterial non-host resistance: interaction of *Arabidopsis* with non-adapted *Pseudomonas syringae* strains.. Physiol Plant.

[6] Loehrer M, Langenbach C, Goellner K, Conrath U, Schaffrath U (2008). Characterization of nonhost resistance of *Arabidopsis* to the Asian soybean rust.. Mol Plant-Microbe Interact.

[7] Huitema E, Vleeshouwers VGAA, Francis DM, Kamoun S (2003). Active defense responses associated with non-host resistance of *Arabidopsis thaliana* to the oomycete pathogen *Phytophthora infestans*.. Mol Plant Pathol.

[8] Gentile A, Deng Z, Malfa SL, Distefano G, Domina F (2007). Enhanced resistance to *Phoma tracheiphila* and *Botrytis cinerea* in transgenic lemon plants expressing a *Trichoderma harzianum* chitinase gene.. Plant Breeding.

[9] Heath MC (2000). Nonhost resistance and nonspecific plant defenses.. Curr Opin Plant Biol.

[10] Mysore KS, Ryu CM (2004). Nonhost resistance: how much do we know?. Trends Plant Sci.

[11] Holub EB, Cooper A (2004). Matrix, reinvention in plants: how genetics is unveiling secrets of non-host disease resistance.. Trends Plant Sci.

[12] Ham JH, Kim MG, Lee SY, Mackey D (2007). Layered basal defenses underlie non-host resistance of Arabidopsis to *Pseudomonas syringae* pv. *phaseolicola*.. Plant J.

[13] Thordal-Christensen H (2003). Fresh insights into processes of nonhost resistance.. Curr Opin Plant Biol.

[14] Fan J, Crooks C, Creissen G, Fairhurst S, Doerner P (2011). *Pseudomonas sax* gene overcome aliphatic isothiocyanate-mediated non-host resistance in *Arabidopsis*.. Science.

[15] Hiruma K, Onozawa-Komori M, Takahashi F, Asakura M, Bednarek P (2010). Entry mode-dependent function of an indole glucosinolate pathway in Arabidopsis for nonhost resistance against anthracnose pathogens.. Plant Cell.

[16] Sanchez-Vallet A, Ramos B, Bednarek P, López G, Piślewska-Bednarek M (2010). Tryptophan-derived secondary metabolites in *Arabidopsis thaliana* confer non-host resistance to necrotrophic *Plectosphaerella cucumerina* fungi.. Plant J.

[17] Zurbriggen MD, Carrillo N, Tognetti VB, Melzer M, Peisker M (2009). Chloroplast-generated reactive oxygen species play a major role in localized cell death during the non-host interaction between tobacco and *Xanthomonas campestris* pv. *vesicatoria*.. Plant J.

[18] Feechan A, Kwon E, Yun BW, Wang Y, Pallas JA (2005). A central role for *S*-nitrosothiols in plant disease resistance.. Proc Natl Acad Sci USA.

[19] Senthil-Kumar M, Mysore KS (2010). Assessing functional role of three water deficit-induced genes in nonhost disease resistance using virus-induced gene silencing in *Nicotiana benthamiana*.. Plant Signal Behav.

[20] Collins NC, Thordal-Christensen H, Lipka V, Bau S, Kombrink E (2003). SNARE-protein-mediated disease resistance at the plant cell wall.. Nature.

[21] Kwon C, Neu C, Pajonk S, Yun HS, Lipka U (2008). Co-option of a default secretory pathway for plant immune responses.. Nature.

[22] Lipka V, Dittgen J, Bednarek P, Bhat R, Wiermer M (2005). Pre- and postinvasion defenses both contribute to nonhost resistnace in Arabidopsis.. Science.

[23] Stein M, Dittgen J, Sanchez-Rodriguez C, Hou BH, Molina A (2006). Arabidopsis PEN3/PDR8, an ATP binding cassette transporter, contributes to nonhost resistance to inappropriate pathogens that enter by direct penetration.. Plant Cell.

[24] Yun BW, Atkinson HA, Gaborit C, Greenland A, Read ND (2003). Loss of actin cytoskeletal function and EDS1 activity, in combination, severely compromises non-host resistance in Arabidopsis against wheat powdery mildew.. Plant J.

[25] Tao Y, Xie Z, Chen W, Glazebrook J, Chang HS (2003). Quantitative nature of Arabidopsis responses during compatible and incompatible interactions with the bacterial pathogen *Pseudomonas syringae*.. Plant Cell.

[26] Navarro L, Zipfel C, Rowland O, Keller I, Robatzek S (2004). The transcriptional innate immune response to flg22. Interplay and overlap with *Avr* gene-dependent defense responses and bacterial pathogenesis.. Plant Physiol.

[27] Felix G, Boller T (2003). Molecular sensing of bacteria in plans. The highly conserved RNA-binding motif RNP-1 of bacterial cold shock proteins is recognized as an elicitor signal in tobacco.. J Biol Chem.

[28] Fliegmann J, Mithöfer A, Wanner G, Ebel J (2004). An ancient enzyme domain hidden in the putative β-glucan elicitor receptor of soybean may play an active part in the perception of pathogen-associated molecular patterns during broad host resistance.. J Biol Chem.

[29] Zipfel C, Kunze G, Chinchilla D, Caniard A, Jones JD (2006). Perception of the bacterial PAMP EF-Tu by the receptor EFR restricts *Agrobacterium*-mediated transformation.. Cell.

[30] Shimizu R, Taguchi F, Marutani T, Inagaki Y, Toyoda K (2003). The DeltafliD mutant of *Pseudomonas syringae* pv. *tabaci*, which secretes flagellin monomers, induces a strong hypersensitive reaction (HR) in non-host tomato cells.. Mol Genet Genomics.

[31] Li X, Lin H, Zhang W, Zou Y, Zhang J (2005). Flagellin induces innate immunity in nonhost interactions that is suppressed by *Pseudomonas syringae* effectors.. Proc Natl Acad Sci USA.

[32] Matsumura K, Tosa Y (1995). The rye mildew fungus carries avirulence genes corresponding to wheat genes for resistance to races of the wheat mildew fungus.. Phytopathol.

[33] Zhao B, Lin X, Poland J, Trick H, Leach J (2005). A maize resistance gene functions against bacterial streak disease in rice.. Proc Natl Acad Sci USA.

[34] Azevedo C, Betsuyaku S, Peart J, Takahashi A, Noël L (2006). Role of SGT1 in resistance protein accumulation in plant immunity.. EMBO J.

[35] Noël LD, Cagna G, Stuttmann J, Wirthmüller L, Betsuyaku S (2007). Interaction between SGT1 and cytosolic/nuclear HSC70 chaperones regulates Arabidopsis immune responses.. Plant Cell.

[36] Shirasu K (2009). The HSP90-SGT1 chaperone complex for NLR immune sensors.. Annu Rev Plant Biol.

[37] Kadota Y, Shirasu K, Guerois R (2010). NLR sensors meet at the SGT1-HSP90 crossroad.. Trends Biochem Sci.

[38] Sun X, Gilroy EM, Chini A, Nurmberg PL, Hein I (2011). *ADS1* encodes a MATE-transporter that negatively regulates plant disease resistance.. New Phyt.

[39] Coemans B, Takahashi Y, Berberich T, Ito A, Kanzaki H (2008). High-throughput *in planta* expression screening identifies an ADP-ribosylation factor (*ARF1*) involved in non-host resistance and *R* gene-mediated resistance.. Mol Plant Pathol.

[40] Hiruma K, Nishiuchi T, Tomoaki K, Bednarek P, Okuno T (2011). *Arabidopsis ENHANCED DISEASE RESISTANCE 1* is required for pathogen-induced expression of plant defensins in nonhost resistance, and acts through interference of *MYC2*-mediated repressor function.. Plant J.

[41] Zhang J, Lu HB, Li XY, Li Y, Cui HT (2010). Effector-triggered and PAMP-triggered immunity differentially contribute to basal resistance to *Pseudomonas syringae*.. Mol Plant-Microbe Interact.

[42] Peart LUR, Sadanandom A, Malcuit I, Moffett P, Brice DC (2002). Ubiquitin ligase-associated protein SGT1 is required for host and nonhost disease resistance in plants.. Proc Natl Acad Sci USA.

[43] Kanzaki H, Saitoh H, Ito A, Fujisawa S, Kamoun S (2003). Cytosolic HSP90 and HSP70 are essential components of INF1-mediated hypersensitive response and non-host resistance to *Pseudomonas cichorii* in *Nicotiana benthamiana*.. Mol Plant Pathol.

[44] Shibata Y, Kawakita K, Takemoto D (2011). *SGT1* and *HSP90* are essential for age-related non-host resistance of *Nicotiana benthamiana* against the oomcete pathogen *Phytophthora infestans*.. Physiol Mol Plant Pathol.

[45] Thomma BP, Nelissen I, Eggermont K, Broekaert WF (1999). Deficiency in phytoalexin production causes enhanced susceptibility of *Arabidopsis thaliana* to the fungus *Alternaria brassicicola*.. Plant J.

[46] Lu M, Tang X, Zhou JM (2001). Arabidopsis *NHO1* is required for general resistance against *Pseudomonas* bacteria.. Plant Cell.

[47] Kang L, Li J, Zhao T, Xiao F, Tang X (2003). Interplay of the Arabidopsis nonhost resistance gene *NHO1* with bacterial virulence.. Proc Natl Acad Sci USA.

[48] Mellersh DG, Heath MC (2003). An investigation into the involvement of defense signaling pathways in components of the nonhost resistance of *Arabidopsis thaliana* to rust fungi also reveals a model system for studying rust fungal compatibility.. Mol Plant-Microbe Interact.

[49] Thomma BPHJ, Eggermont K, Penninckx IAMA, Mauch-Mani B, Vogelsang B (1998). Separate jasmonate-dependent and salicylate-dependent defense-response pathways in Arabidopsis are essential for resistance to distinct microbial pathogens.. Proc Natl Acad Sci USA.

[50] Knoester M, Van Loon LC, Van Den Heuvel J, Hennig J, Bol JF (1998). Ethylene-insensitive tobacco lacks nonhost resistance against soil-borne fungi.. Proc Natl Acad Sci USA.

[51] Wen Y, Wang W, Feng J, Luo MC, Tsuda K (2011). Identification and utilization of a sow thistle powdery mildew as a poorly adapted pathogen to dissect post-invasion non-host resistance mechanisms in Arabidopsis.. J Exp Botany.

[52] Apel K, Hirt H (2004). Reactive oxygen species: metabolism, oxidative stress, and signal transduction.. Annu Rev Plant Biol.

[53] Hückelhoven R, Kogel KH (2003). Reactive oxygen intermediates in plant-microbe interactions: who is who in powdery mildew resistance?. Planta.

[54] Mehdy MC (1994). Active oxygen species in plant defense against pathogens.. Plant Physiol.

[55] Nimchuk Z, Eulgem T, Holt BF, Dangl JF (2003). Recognition and response in the plant immune system.. Annu Rev Genet.

[56] Grant JJ, Yun BW, Loake GJ (2000). Oxidative burst and cognate redox signalling reported by luciferase imaging: identification of a signal network that functions independently of ethylene, SA and Me-JA but is dependent on MAPKK activity.. Plant J.

[57] Asai T, Tena G, Plotnikova J, Willmann MR, Chiu WL (2002). MAP kinase signaling cascade in Arabidopsis innate immunity.. Nature.

[58] Uknes S, Mauch-Mani B, Moyer M, Potter S, Williams S (1992). Acquired resistance in Arabidopsis.. Plant Cell.

[59] Wildermuth MC, Dewdney J, Wu G, Ausubel FM (2001). Isochorismate synthase is required to synthesize salicylic acid for plant defence.. Nature.

[60] Hauck P, Thilmony R, He SY (2003). A *Pseudomonas syringae* type III effector suppresses cell wall-based extracellular defense in susceptible *Arabidopsis* plants.. Proc Natl Acad Sci USA.

[61] Adams-Phillips L, Briggs AG, Bent AF (2010). Disruption of poly(ADP-ribosyl)ation mechanisms alters responses of Arabidopsis to biotic stress.. Plant Physiol.

[62] Truman W, Zabala MT, Grant M (2006). Type III effectors orchestrate a complex interplay between transcriptional networks to modify basal defence responses during pathogenesis and resistance.. Plant J.

[63] Heath MC (2001). Non-host resistance to plant pathogens: Nonspecific defense or the result of specific recognition events?. Physiol Mol Plant Pathol.

[64] Kamoun S (2001). Nonhost resistance to *Phytophthora*: novel prospects for a classical problem.. Curr Opin Plant Biol.

[65] Nürnberger T, Brunner F, Kemmerling B, Piater L (2004). Innate immunity in plants and animals: striking similarities and obvious differences.. Immunol Rev.

[66] Gómez-Gómez L, Boller T (2002). Flagellin perception: a paradigm for innate immunity.. Trends Plant Sci.

[67] Nürnberger T, Lipka V (2005). Non-host resistance in plants: new insights into an old phenomenon.. Mol Plant Pathol.

[68] Bindschedler LV, Dewdney J, Blee KA, Stone JM, Asai T (2006). Peroxidase-dependent apoplastic oxidative burst in *Arabidopsis* required for pathogen resistance.. Plant J.

[69] Soylu S, Brown I, Mansfied JW (2005). Cellular reactions in *Arabidopsis* following challenge by strains of *Pseudomonas syringae*: from basal resistance to compatibility.. Physiol Mol Plant Pathol.

[70] Torres MA, Jones JDG, Dangl JL (2005). Pathogen-indueced, NADPH oxidase-derived reactive oxygen intermediates suppress spread of cell death in *Arabidopsis thaliana*.. Nat Genetics.

[71] Jacobs AK, Lipka V, Burton RA, Panstruga R, Strizhov N (2003). An Arabidopsis callose synthase, GSL5, is required for wound and papillary callose formation.. Plant Cell.

[72] Glazebrook J (2005). Contrasting mechanisms of defense against biotrophic and necrotrophic pathogens.. Annu Rev Phytopathol.

[73] Hardham AR, Jones DA, Takemoto D (2007). Cytoskeleton and cell wall function in penetration resistance.. Curr Opin Plant Biol.

[74] Hao P, Liu C, Wang Y, Chen R, Tang M (2008). Herbivore-induced callose deposition on the sieve plates of rice: an important mechanism for host resistance.. Plant Physiol.

[75] Nishimura MT, Stein M, Hou BH, Vogel JP, Edwards H (2003). Loss of a callose synthase results in salicylic acid-dependent disease resistance.. Science.

[76] van Wees SCM, Glazebrook J (2003). Loss of non-host resistance of *Arabidopsis NahG* to *Pseudomonas syringae* pv. *phaseolicola* is due to degradation products of salicylic acid.. Plant J.

[77] Narusaka Y, Narusaka M, Seki M, Ishida J, Shinozaki K (2005). Cytological and molecular analyses of non-host resistance of *Arabidopsis thaliana* to *Alternaria alternata*.. Mol Plant Pathol.

[78] van Baarlen P, Woltering EJ, Staats M, van Kan JAL (2007). Histochemical and genetic analysis of host and non-host interactions of *Arabidopsis* with three *Botrytis* species: an important role for cell death control.. Mol Plant Pathol.

[79] Niks RE (1988). Nonhost plant species as donors for resistance to pathogens with narrow host range. II. Concepts and evidence on the genetic basis of nonhost resistance.. Euphytica.

[80] Wan AM, Zhao ZH, Chen XM, He ZH, Jin SL (2004). Wheat stripe rust epidemics and virulence of *Puccinia striiformis* f. sp. *tritici*.. Plant Dis.

[81] Cao H, Glazebrook J, Clark JD, Volko S, Dong X (1997). The Arabidopsis *NPR1* gene that control systemic acquired resistance encodes a novel protein containing ankyrin repeats.. Cell.

[82] Aarts N, Metz M, Holub E, Staskawicz BJ, Daniels MJ (1998). Different requirements for EDS1 and NDR1 by disease resistance genes define at least two *R* gene-mediated signaling pathways in *Arabidopsis*.. Proc Natl Acad Sci USA.

[83] Nawrath C, Heck S, Parinthawong N, Métraux JP (2002). EDS5, an essential component of salicylic acid-dependent signaling for disease resistance in Arabidopsis, is a member of the MATE transporter family.. Plant Cell.

[84] Volko SM, Boller T, Ausubel FM (1998). Isolation of new *Arabidopsis* mutants with enhanced disease susceptibility to *Pseudomonas syringae* by direct screening.. Genetics.

[85] Zhou N, Tootle TL, Glazebrook J (1999). Arabidopsis *PAD3*, a gene required for camalexin biosynthesis, encodes a putative cytochrome P450 monooxygenase.. Plant Cell.

[86] Jirage D, Tootle T, Reuber TL, Frost LN, Feys BJ (1999). *Arabidopsis thaliana PAD4* encodes a lipase-like gene that is important for salicylic acid signaling.. Proc Natl Acad Sci USA.

[87] Century KS, Shapiro AD, Repetti PP, Dahlbeck D, Holub E (1997). *NDR1*, a pathogen-induced component, required for *Arabidopsis* disease resistance.. Science.

[88] Guzmán P, Ecker JR (1990). Exploiting the triple response of Arabidopsis to identify ethylene-related mutants.. Plant Cell.

[89] Staswick PE, Tiryaki I, Rowe ML (2002). Jasmonate response locus *JAR1* and several related *Arabidopsis* genes encode enzymes of the firefly luciferase superfamily that show activity on jasmonic, salicylic, and indole-3-acetic acids in an assay for adenylation.. Plant Cell.

[90] Clarke JD, Liu Y, Klessig DF, Dong X (1998). Uncoupling *PR* gene expression from NPR1 and bacterial resistance: characterization of the dominant Arabidopsis *cpr6-1* mutant.. Plant Cell.

[91] Katagiri F, Thilmony R, He SY, Somerville CR, Meyerowitz EM (2002). The Arabidopsis-*Pseudomonas syringae* interaction.. The Arabidopsis book.

[92] da Silva ACR, Ferro JA, Reinach FC, Farah CS, Furlan LR (2002). Comparison of the genomes of two *Xanthomonas* pathogens with different host specificities.. Nature.

[93] Adam L, Somerville SC (1996). Genetic characterization of five powdery mildew disease resistance loci in *Arabidopsis thaliana*.. Plant J.

[94] Weigel D, Glazebrook J (2002). Arabidopsis: a laboratory manual.

[95] Cao H, Bowling SA, Gordon S, Dong X (1994). Characterization of an Arabidopsis mutant that is nonresponsive to inducers of systemic acquired resistance.. Plant Cell.

[96] DeFraia CT, Zhang X, Mou Z (2010). Elongator subunit 2 is an accelerator of immune responses in *Arabidopsis thaliana*.. Plant J.

[97] Verberne MC, Brouwer N, Delbianco F, Linthorst HJ, Bol JF (2002). Method for the extraction of the volatile compound salicylic acid from tobacco leaf material.. Phytochem Anal.

